# Mortality and morbidity caused by pneumonia in children and adolescents are associated with socioeconomic and urban conditions in São Paulo, Brazil

**DOI:** 10.1590/1984-0462/2026/44/2025214

**Published:** 2026-06-22

**Authors:** William Cabral-Miranda, Celina de Almeida Lamas, Cauê Beloni, Karen Regina Amato Samos, Fátima Rodrigues Fernandes, Gustavo Falbo Wandalsen

**Affiliations:** a Instituto Pensi, Fundação José Luiz Setúbal, São Paulo, SP, Brazil.; b Hospital do Servidor Público Municipal, São Paulo, SP, Brazil.

**Keywords:** Geographic information systems, Socioeconomic factors, Urban population, Public health, Sistemas de informação geográfica, Fatores socioeconômicos, População urbana, Saúde pública

## Abstract

**Objective:**

The aim of this study was to investigate the association between pneumoniarelated mortality and morbidity and urban, environmental, and socioeconomic conditions in the administrative districts of São Paulo, Brazil.

**Methods:**

We conducted an ecological study using aggregated data from 2010 to 2020 across 96 administrative districts in ã Paulo. Mortality and hospitalization data for individuals up to 19 years old were analyzed in relation to the Geographic Index of the Socioeconomic Context for Health and Social Studies (GeoSES), housing standards, and urban conditions. Spatial analysis involved estimating relative risk using SaTScan, and Ordinary Least Squares (OLS) and Geographically Weighted Regression (GWR) were applied to assess spatial associations.

**Results:**

Between 2010 and 2020, 1486 deaths and 1,56,112 hospitalizations due to pneumonia were recorded among individuals up to 19 years old. Mortality was higher among males (54.7%) and displayed spatial clustering. Mortality rates were negatively associated with the proportion of high-standard residences and the GeoSES index and positively associated with the presence of rapid-transit roadways. The GWR model outperformed the OLS model (adjusted R2=0.44 vs. 0.40) for mortality. Hospitalization rates were more uniformly distributed and showed a weaker association with socioeconomic indicators (adjusted R2=0.17 for OLS).

**Conclusions:**

Pneumonia mortality among children and adolescents in São Paulo is spatially associated with socioeconomic and urban conditions. These findings underscore the need for geographically targeted public health interventions that address urban planning and social inequalities in disease prevention strategies.

## RESUMO

**Objetivo:**

Investigar a associação entre mortalidade e morbidade por pneumonia e condições urbanas, ambientais e socioeconômicas nos distritos administrativos de São Paulo, Brasil.

**Métodos:**

Estudo ecológico com dados agregados de 2010 a 2020, abrangendo 96 distritos administrativos. Foram analisados óbitos e internações por pneumonia em indivíduos de zero a 19 anos, com relação ao Índice Geográfico do Contexto Socioeconômico para Estudos de Saúde e Sociais (GeoSES), padrões habitacionais e condições urbanas. A análise espacial utilizou o software SaTScan para estimativa de risco relativo, e modelos de regressão linear simples (OLS) e ponderada geograficamente (GWR) foram aplicados para avaliar associações espaciais.

**Resultados:**

No período, ocorreram 1.486 óbitos e 156.112 internações por pneumonia entre indivíduos de até 19 anos. A mortalidade foi maior no sexo masculino (54,7%) e apresentou aglomeração espacial. As taxas de mortalidade mostraram associação negativa com a proporção de residências de alto padrão e com o índice GeoSES e associação positiva com a presença de vias expressas. O modelo GWR teve melhor desempenho do que o OLS (R2 ajustado=0,44 vs. 0,40). As internações foram mais uniformemente distribuídas e menos associadas a indicadores socioeconômicos (R2 ajustado=0,17 para OLS).

**Conclusões:**

A mortalidade por pneumonia em crianças e adolescentes em São Paulo está associada ao padrão socioeconômico e à desigualdade na condição urbana. Os resultados reforçam a necessidade de políticas públicas com enfoque territorial, que integrem planejamento urbano e equidade social nas estratégias de prevenção de doenças respiratórias.

## INTRODUCTION

 Pneumonia is an infection that settles in the lungs caused by the penetration of infectious agents. In adults, it can be caused by several factors that can affect the immune system’s capacity and, consequently, the defense of the respiratory system. Pneumonia remains a leading cause of morbidity and mortality among children and adolescents under 18 years of age, particularly in low- and middle-income countries. In 2019, it accounted for approximately 740,180 deaths among children under five, representing about 14% of all deaths in this age group.^1,2^ Among children aged 5–9 years, pneumonia continues to be a significant cause of death, resulting in tens of thousands of preventable fatalities globally.^2^ While specific data for adolescents aged 10–19 years are limited, respiratory infections, including pneumonia, contribute to the more than 3,000 adolescent deaths occurring daily worldwide.^3^ The highest burden is observed in sub-Saharan Africa and South Asia, where access to preventive measures and appropriate care remains limited.^1 ,2^ Progress has been made in preventing pneumonia, especially through the development of vaccines such as those for *Haemophilus influenzae* type B and *Streptococcus pneumoniae*.^4^ Although intervention through immunization is the most effective initiative, it does not prevent all episodes of pneumonia.^5^ Understanding how environmental and socioeconomic factors are associated with this disease can contribute to the epidemiological comprehension of pneumonia, serving as a reinforced instrument in the implementation of preventive measures, such as immunization.^6^

 Studies have suggested that infectious diseases are associated with socioeconomic conditions of the populations, leading to a dangerous cycle for vulnerable children. There is evidence that poor living conditions, crowded housing, basic sanitation, and inadequate water distribution are associated with pneumonia, where children are repeatedly exposed to viral and bacterial infections.^7^

 Family income is another basic element in assessing health conditions and plays a crucial role in evaluating the availability of health resources.^8^ Mortality from preventable causes is mainly concentrated in populations with the lowest social strata.^9^ Families with income below the minimum wage have an increased risk of hospitalization for pneumonia. A study conducted in Salvador, northeastern Brazil, showed that poorer children were hospitalized more frequently and for longer periods with more severe conditions and higher death rates, while children of higher socioeconomic status had milder clinical manifestations of pneumonia.^10^ The education level of family members, particularly maternal education, is another factor closely associated with the health and survival of children and young people. More educated mothers may better understand how to prevent and care for their child, when to seek help, and the importance of following medical recommendations.^11^ Moreover, the number of people living in the same residence can increase the risk of infection and has been highlighted as an important factor in the transmission of respiratory diseases due to greater interpersonal contact.^10^

 In addition to these issues, environmental conditions may be associated with hospitalizations caused by pneumonia, asthma, and chronic obstructive pulmonary diseases.^12^ Studies highlight that poor air quality, high levels of particulate matter, and exposure to environmental stressors contribute to the incidence and severity of these conditions.^13^ Recent evidence suggests that living close to green areas is associated with a decreased risk of mortality, including respiratory diseases, as well as other health conditions.^14^ Green areas can reduce exposure to pollutants and the intensity of urban heat islands,^15^ filtering, dispersing, and blocking air pollutants, and creating healthier microenvironments. Furthermore, access to green spaces has been associated with increased opportunities for physical activity, social interaction, and stress reduction, all of which contribute positively to overall health and resilience against disease. 

 Thus, in this study, we analyzed the association between the relative risks of mortality caused by pneumonia in children and young people up to 19 years and the environmental, urban, and socioeconomic conditions in the administrative districts of São Paulo, Brazil, from 2010 to 2020. 

## METHOD

 The research was carried out in the 96 administrative districts of the municipality of São Paulo, the capital of the state of São Paulo, located in southeastern Brazil. São Paulo is the most populous city in Brazil, and its municipality population corresponds to 11,253,503 inhabitants, based on the last demographic census in 2010, with an estimated population of 12,252,023 inhabitants for the year 2019. The population studied comprised age groups from <1 to 19 years old, totaling 3,178,893 citizens.^16^

 This is an ecological study in which pneumonia mortality and morbidity data and explanatory variables were aggregated and analyzed at the municipal administrative district level in the municipality of São Paulo. Mortality and morbidity data related to diseases of the respiratory system were used for the period from 2010 to 2020. These data are available and free of charge from the Sistema de Informações sobre Mortalidade — SIM/PRO-AIM–CEInfo–SMS-SP,^17^ from the demographic census performed by the Instituto Brasileiro de Geografia e Estatística (IBGE).^16^ The study focused on pneumonia cases classified under the International Classification of Diseases, 10th Revision (ICD-10), including: J12 — Viral pneumonia, not elsewhere classified; J13 — Pneumonia due to *Streptococcus pneumoniae*; J14 — Pneumonia due to *Haemophilus influenzae*; J15 — Bacterial pneumonia, not elsewhere classified; J16 — Pneumonia due to other infectious organisms, not elsewhere classified; J17 — Pneumonia in diseases classified elsewhere; J18 — Pneumonia, organism unspecified; and P23 — Congenital pneumonia. 

 The environmental and urban variables were related to street trees (number/km^2^), slum areas (km^2^), and the proportions of medium- and high-standard residences and low-standard residences (refers to the of.ficial classification of residential buildings established by the municipality of São Paulo for fiscal and cadastral purposes). This classification is based on construction characteristics and building standards, highways (km), rapid transit routes (km), the Geographic Index of the Socioeconomic Context for Health and Social Studies (GeoSES), and the number of healthcare facilities per 100 inhabitants. All variables were calculated and aggregated by administrative districts. These data were obtained through Geosampa.^18^

 GeoSES was developed by Barrozo et al.,^19^ primarily based on the analysis of the 2010 demographic sample from the IBGE census.^16^ The GeoSES is a composite indicator designed to assess socioeconomic inequalities in health research across Brazil. It encompasses seven key dimensions: education, mobility, poverty, wealth, income, segregation, and deprivation of resources and services. This multidimensional approach enables the index to capture both material and social aspects of living conditions, providing a comprehensive measure of socioeconomic status. 

 The cartographic bases of the 96 administrative districts of São Paulo, as well as the environmental and urban variables, were obtained from the GeoSampa.^18^ To represent the relative risks and spatial clustering, we used the dasymetric cartographic base standardized by Barrozo et. al.,^19^ using the choroplethic and chorochromatic representation methods, respectively. ArcGis 10.4 was used to perform operations and represent variables. 

 Mortality and hospitalization rates were calculated by dividing the number of pneumonia-related deaths or hospital admissions among individuals aged 0–19 years by the corresponding population in the municipality of São Paulo. The resulting values were multiplied by 100,000 to obtain annual rates per 100,000 inhabitants. Population denominators were derived from the IBGE. 

 Relative risks (RRs) were estimated using the SaTScan software, which applies the spatial scan statistic^20^ to identify geographic clusters of pneumonia-related mortality and hospitalizations. The RR for each cluster was calculated as the ratio of observed to expected cases within the cluster, using the underlying population aged 0–19 years in each municipality district. This approach allows identification of areas where event occurrence is significantly higher (RR>1) or lower (RR<1) than expected under the null hypothesis of random spatial distribution. Statistical significance was assessed using 999 Monte Carlo replications, and clusters with p<0.05 were considered significant.^20^ Because the analysis was ecological and based on aggregated population-level data, no individual-level confounding factors (such as comorbidities or socioeconomic conditions) were included in the RR calculation; however, the SaTScan model directly incorporated population denominators to adjust for differences in population size across areas. 

 Ordinary least squares (OLS) estimates the unknown parameters in a linear regression model by minimizing the sum of the squares of the differences between the observed and predicted values as a function of the explanatory variables. Geographically weighted regression (GWR) extends the OLS approach by incorporating spatial dependence through a kernel bandwidth.^21^ All analyses were performed on ArcGIS 10.4. 

## RESULTS

 Between 2010 and 2020, a total of 1,486 pneumonia-related deaths were recorded among individuals aged 19 years or younger in the municipality of São Paulo. Of these, 814 deaths (54.7%) occurred among males, while 672 (45.3%) were among females. This figure corresponds to an average annual mortality rate of 4.2 per 100,000 inhabitants. During the same period and within the same population group, a total of 156,112 hospitalizations for pneumonia were reported. This represents an average annual hospitalization rate of 446.4 per 100,000 inhabitants. Among these cases, 83,469 (53.4%) occurred in males, whereas 72,646 (46.6%) occurred in females (Table 1). 

**Table 1.** Pneumonia-related mortality and hospital admissions among population aged 0–19 years in São Paulo (SP), 2010–2020.

Outcome	Total (n)	Male n (%)	Female n (%)	Average annual rate (per 1,00,000)
Mortality	1,486	814 (54.7)	672 (45.3)	4.2
Morbidity	156,112	83,469 (53.4)	72,646 (46.6)	446.4

**Figure 1:**
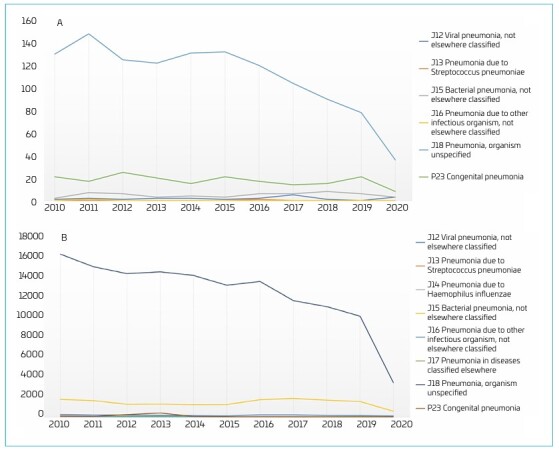
(A) Specific causes of mortality from pneumonia, (B) Specific causes of hospitalization due to pneumonia, population aged 0–19 years in the municipality of São Paulo, 2010–2020.

 An analysis of the specific causes of death revealed a predominance of cases classified as unspecified viral pneumonia (ICD-10: J12), particularly between 2010 and 2015, followed by a continuous downward trend from 2016 onward. Other categories, including pneumonia due to *Streptococcus pneumoniae* (J13), *Haemophilus influenzae* (J14), unspecified bacterial pneumonia (J15), and congenital pneumonia (P23), exhibited significantly lower frequencies and remained relatively stable throughout the historical series. A sharp reduction in total deaths was observed in 2020 (Figure 1A). 

 As observed with mortality, the highest proportion of hospitalizations was associated with cases classified as pneumonia of unspecified etiology (J18). This category showed consistently high numbers over the entire period, with a gradual decline from 2010 to 2019, followed by a more pronounced drop in 2020. Other etiological subtypes — including viral pneumonia (J12), bacterial pneumonia (J15), and those caused by *Streptococcus pneumoniae* (J13) and *Haemophilus influenzae* (J14) — accounted for smaller, relatively stable proportions throughout the series (Figure 1B). 

 These data demonstrate a consistent pattern of decline in both mortality and hospital morbidity due to pneumonia among individuals up to 19 years old in the municipality of São Paulo between 2010 and 2020. 

**Figure 2:**
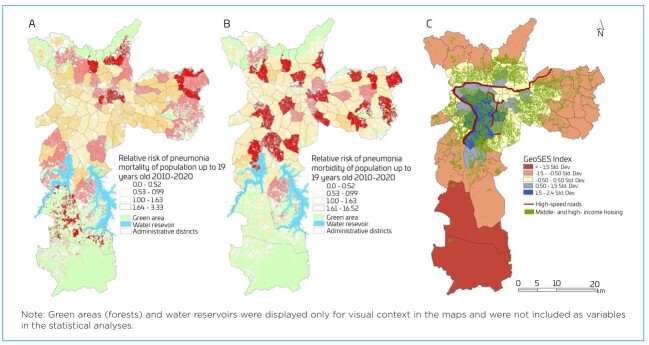
Relative risk of pneumonia outcomes for the entire study period (2010–2020) among individuals aged 0–19 years in the municipality of São Paulo, by districts: (A) mortality, (B) morbidity, and (C) significant socioeconomic and urban explanatory variables (GeoSES index, rapid-transit routes, and proportion of medium- and high-standard residences).

 The spatial modeling results suggest a distinct pattern between pneumonia-related morbidity (Figure 2A) and mortality (Figure 2B) across the municipality of São Paulo. Hospital admissions appear to occur relatively uniformly throughout the city, with no strong spatial clustering or localized associations, indicating that exposure to risk factors for pneumonia morbidity may be broadly distributed and consistent across administrative districts. In contrast, mortality from pneumonia exhibits a more defined spatial pattern, with significant associations with socioeconomic and urban conditions. Figure 2C summarizes the socioeconomic and urban condition variables that were statistically associated with the child health outcomes in this study. 

 A regression analysis was conducted to assess the association between pneumonia-related mortality in individuals up to 19 years old and selected socioeconomic indicators and urban factors across administrative districts in the municipality of São Paulo. Two models were employed: OLS and GWR. 

 In the OLS model, the adjusted R^2^ was 0.40, the Akaike Information Criterion (AIC) was 105.07, and the model was statistically significant (p<0.001). Mortality rates were negatively associated with the proportion of medium- and high-standard residences and with the GeoSES index, indicating lower mortality in districts with better socioeconomic conditions. A positive association was observed between mortality and the total length of rapid transit roadways in each district (Table 2). 

**Table 2.** Statistical analysis of pneumonia mortality in the population aged 0–19 years in São Paulo, 2010–2020, and significant explanatory variables.

OLS Regression (Mortality)		
Model	Adjusted R^2^	AICs
(−) Medium- and high standard housing*	0.40	105.07
(+) High-speed transit routes*	**Geographically Weighted Regression**	
(−) GeoSES Index*	0.44	101.95

OLS: ordinary least squares; AIC: Akaike Information Criterion. Notes: Statistical models included the following explanatory variables: density of street trees (number/km2), slum areas (km2), proportion of medium- and high-standard residences, proportion of low-standard residences, length of highways (km), length of rapid-transit routes (km), GeoSES index, and number of healthcare facilities per 100 inhabitants. Only statistically significant associations are shown in the table. No individual-level confounders were included, since the analysis was ecological and based on aggregated district-level data.

* p<0.001

 The GWR model demonstrated improved performance, with an adjusted R^2^ of 0.44 and a lower AIC of 101.95. The inclusion of spatially varying coefficients improved the fit to the data, indicating that the associations between the predictors and pneumonia mortality varied across geographic locations within the city. These results indicate spatial variability in the influence of socioeconomic and infrastructural factors on pneumonia mortality, with the GWR model offering a more accurate representation of local associations than the global OLS approach. 

 To assess the spatial determinants of pneumonia-related morbidity (Table 3), measured through hospital admission rates among individuals up to 19 years old in the municipality of São Paulo. In the OLS model, the adjusted R^2^ was 0.17, indicating a modest explanatory power, and the AIC was 460.87. Two variables were statistically significant: the length of rapid-transit roadways showed a negative association with pneumonia hospitalizations (p<0.001), suggesting that districts with greater access to high-speed transit routes had lower rates of hospital admissions. The GeoSES index was positively associated with morbidity (p<0.05), indicating that hospitalizations were more frequent in districts with higher socioeconomic status. 

**Table 3.** Statistical analysis of pneumonia morbidity in the population aged 0–19 years in São Paulo, 2010–2020, and significant explanatory variables.

OLS Regression (Mortality)		
Model	Adjusted R^2^	AICs
(−) High-speed transit routes*	0.17	460.87
(+) GeoSES Index†	**Geographically Weighted Regression**	
	0.16	460

OLS: ordinary least squares; AIC: Akaike Information Criterion.Notes: Statistical models included the following explanatory variables: density of street trees (number/km2), slum areas (km2), proportion of medium- and high-standard residences, proportion of low-standard residences, length of highways (km), length of rapid-transit routes (km), GeoSES index, and number of healthcare facilities per 100 inhabitants. Only statistically significant associations are shown in the table. No individual-level confounders were included, since the analysis was ecological and based on aggregated district-level data.

* p<0.001

† p<0.05

 The GWR model resulted in a slightly lower AIC (460.00) but did not improve the adjusted R^2^, which was 0.16. This suggests that spatial variation in the associations was minimal or that the covariates included in the model did not exhibit strong non-stationarity across the city. 

 Analysis of spatial distribution patterns revealed significant associations between pneumonia-related outcomes and socioeconomic and urban variables across administrative districts in São Paulo. 

 Regarding mortality, the relative risk of death due to pneumonia was 74.74% higher in districts characterized by lower GeoSES indices, indicating a strong association between lower socioeconomic index and increased mortality. Conversely, districts with a higher proportion of medium- and high-standard housing exhibited a 45.09% lower relative risk of pneumonia-related mortality. Additionally, a 12.07% increase in relative risk was observed in districts with a greater extent of rapid-transit routes, suggesting potential environmental or traffic-related exposure effects. 

 In contrast, morbidity presented a different spatial pattern. Hospital admissions for pneumonia were 21.89% more frequent in districts with higher GeoSES indices, indicating greater detection, access to health services, or healthcare-seeking behavior in more affluent areas. Notably, districts with fewer kilometers of rapid-transit roadways exhibited a 92.46% higher relative risk of pneumonia-related hospitalizations, possibly reflecting barriers to healthcare access or delayed care in areas with less infrastructural connectivity. 

## DISCUSSION

 The findings of this study reveal a clear temporal and spatial decline in pneumonia-related mortality and morbidity among individuals aged 0–19 years in the municipality of São Paulo between 2010 and 2020. Despite the overall reduction in deaths and hospitalizations, important disparities persist across administrative districts, particularly in relation to socioeconomic and infrastructural conditions. Mortality was disproportionately higher in areas marked by lower socioeconomic status, as indicated by the GeoSES index, and in districts with fewer medium- and high-standard housing, suggesting the enduring influence of social vulnerability on health outcomes. In addition, a positive association between mortality and the extent of high-speed transit routes points to potential environmental factors, possibly linked to pollution exposure. In contrast, morbidity exhibited a different spatial dynamic: higher hospital admission rates were observed in more affluent districts. Together, these results highlight the complex interplay between urban conditions, socioeconomic context, and pediatric respiratory health. 

 Geographic disparities in pneumonia mortality have been previously observed at both regional and intra-municipal levels. On a citywide scale, higher mortality rates are often concentrated in poorer regions, as shown when municipalities are stratified by low, medium, and high socioeconomic status.^22^ However, similar disparities persist within individual municipalities, reflecting internal spatial inequalities.^7,23^ For example, Andrade et al.^24^ analyzed the spatial distribution of pneumonia cases in children across the administrative districts of Goiânia, Brazil, and identified clusters in specific areas. Likewise, Dias et al.^25^ reported a non-random spatial pattern of pneumonia-related hospitalizations in northern Portugal, highlighting significant spatial dependence and emphasizing the need to explore the underlying causes of disease distribution. These findings are consistent with our study, which found that the relative risk of pneumonia-related mortality among children and youth varied across São Paulo, with some districts showing lower-than-expected risks and others displaying substantially elevated risks. 

 Schuck-Paim et al.^22^ further demonstrated that poverty, malnutrition, and low levels of education are significantly linked to increased pneumonia risk. In our analysis, we used the GeoSES index — a comprehensive indicator encompassing education, mobility, poverty, material deprivation, wealth, income, and segregation — to examine socioeconomic disparities across São Paulo’s districts. Our findings revealed a significant negative association between GeoSES and pneumonia relative risk in individuals aged 0–19 years, suggesting that worse socioeconomic conditions are associated with a higher risk of pneumonia-related mortality. 

 Epidemiological evidence also indicates a role for traffic-related air pollution in respiratory infections among children. Vieira et al.^26^ identified traffic emissions as a risk factor, while Xial et al.^27^ reported significant associations between secondary pollutants (ozone and particulate matter) and increased pneumonia risk in Georgia (2002–2008). Jedrychowski et al.^28^ found an association between prenatal exposure to PM2.5 and pneumonia in Krakow, Poland. Kennedy et al.^29^ observed modest associations between traffic emissions and respiratory diseases. Although the associations in our study were positive, the confidence intervals were wide, and the effect sizes were relatively small. Notably, Girguis et al.^30^ found increased risk of respiratory illness in children living near high-traffic areas, though no direct association with PM2.5 alone. Despite these mixed findings, we observed that districts with a high density of rapid transit routes had significantly elevated pneumonia mortality, warranting further investigation into factors beyond pollution alone. 

 From a general perspective, our results indicate that while certain GeoSES indices and the extent of rapid-transit routes are associated with pneumonia morbidity, their overall explanatory power is limited. Moreover, the spatial heterogeneity of these associations was not strongly pronounced, suggesting the presence of additional, unmeasured determinants that may contribute to the spatial variation in pneumonia outcomes. These findings underscore the complex interplay among social determinants, urban conditions, and respiratory infection-related health outcomes. They point to the need for geographically targeted public health strategies that incorporate both environmental exposures and structural inequalities in healthcare access. Although pneumonia incidence may be broadly distributed, the likelihood of mortality from the disease appears to be shaped by systemic inequities, such as substandard housing, low income, and inadequate infrastructure, which may influence disease severity, delay timely access to care, or limit the effectiveness of treatment interventions. 

 This study presents limitations inherent to its ecological design and to the use of aggregated data at the administrative district level. First, the use of aggregated data precludes individual-level analysis, which may lead to ecological fallacy, that is, incorrect inferences about individuals based on group-level data. Furthermore, the socioeconomic and environmental indicators employed may not fully capture the complexity and variability of living conditions within each district, particularly in areas with high internal heterogeneity. 

 In conclusion, this study identified significant associations between pneumonia-related mortality in individuals aged 0–19 years and the socioeconomic and infrastructural characteristics of urban areas in São Paulo. The results show that mortality was higher in administrative districts marked by socioeconomic deprivation, as reflected by lower GeoSES indices and a lower prevalence of medium- and high-standard housing. Additionally, districts with greater coverage of rapid transit roadways exhibited increased mortality risk, suggesting the possible influence of environmental exposures. In contrast, hospitalizations for pneumonia were more uniformly distributed across the municipality. They were more frequent in districts with higher GeoSES indices and fewer kilometers of rapid-transit roadways. This pattern may be driven by determinants not captured by the variables included in our models. The identification of these spatial patterns and their associated variables provides a valuable basis for guiding public health actions to prevent and control pneumonia. Targeted strategies that address structural inequalities and improve access to timely healthcare may help reduce the disease burden among vulnerable children and adolescents. These findings emphasize the importance of incorporating geographic and social context into health planning and policy decision-making. 

## Data Availability

The database that originated the article is available with the corresponding author
